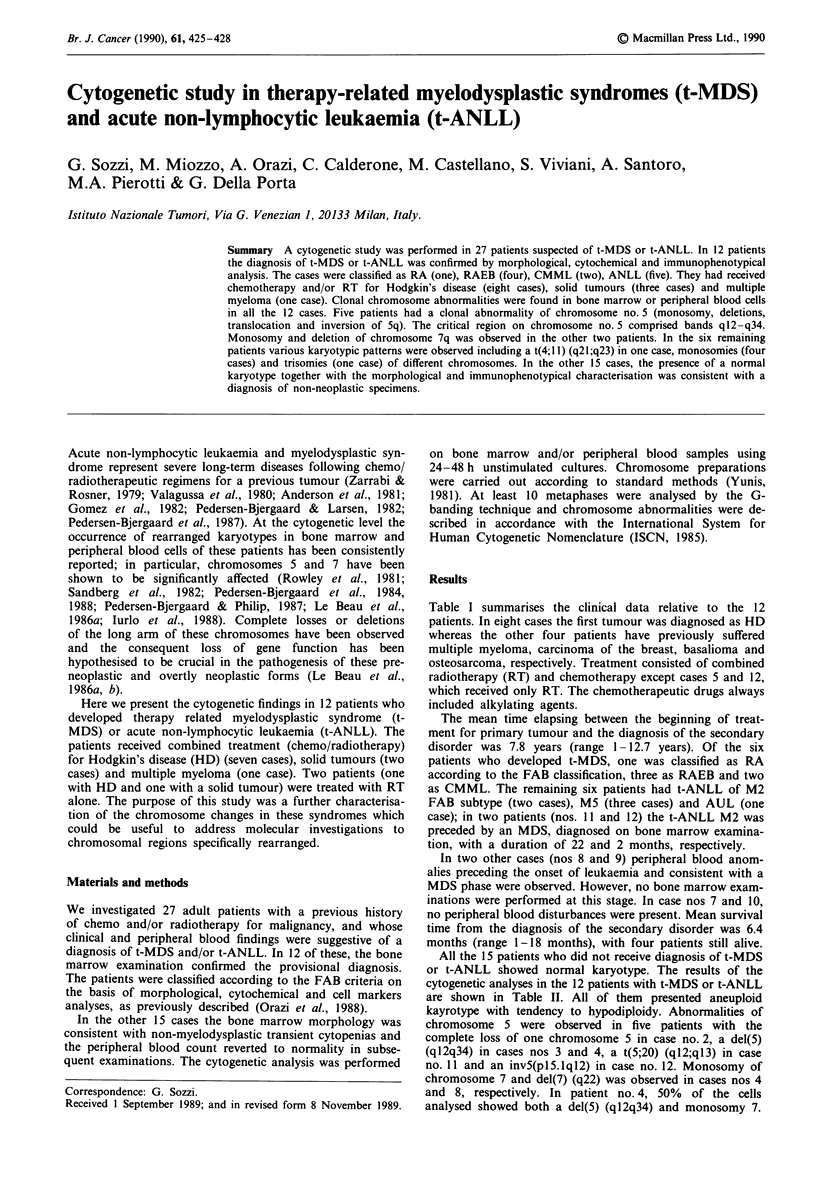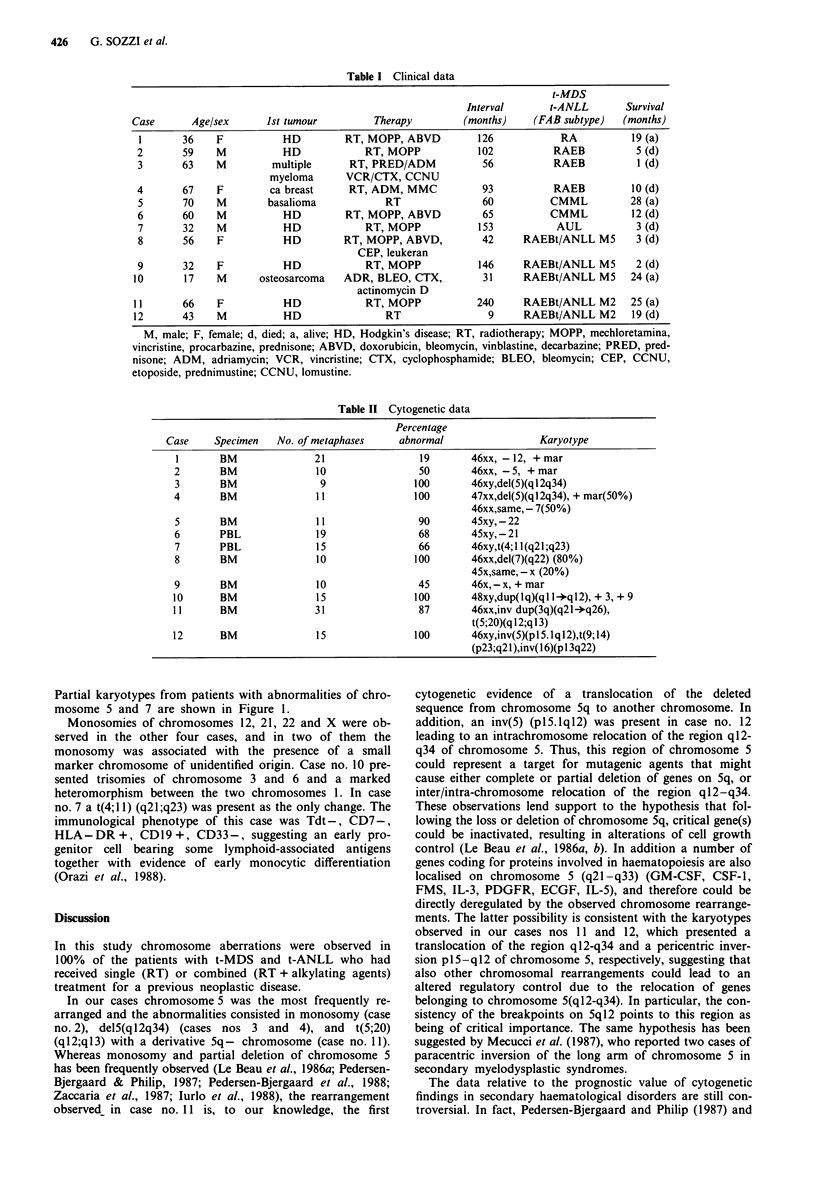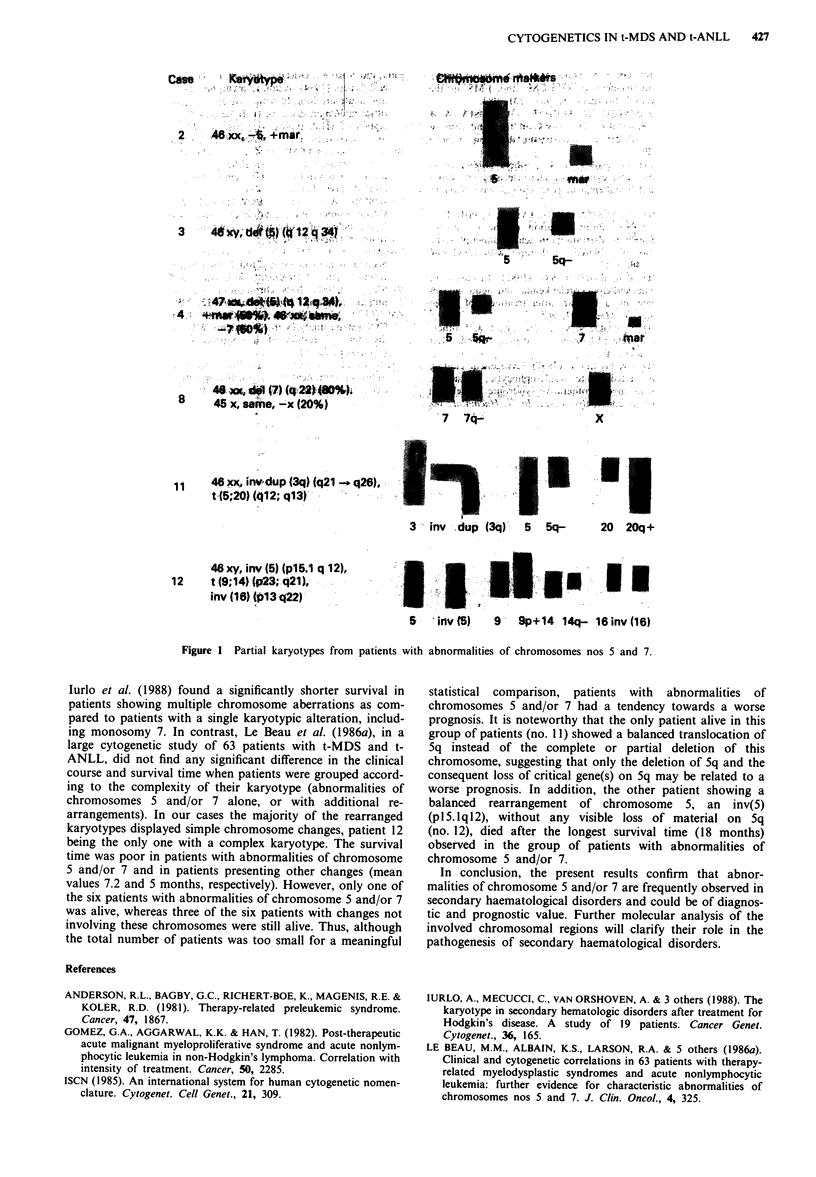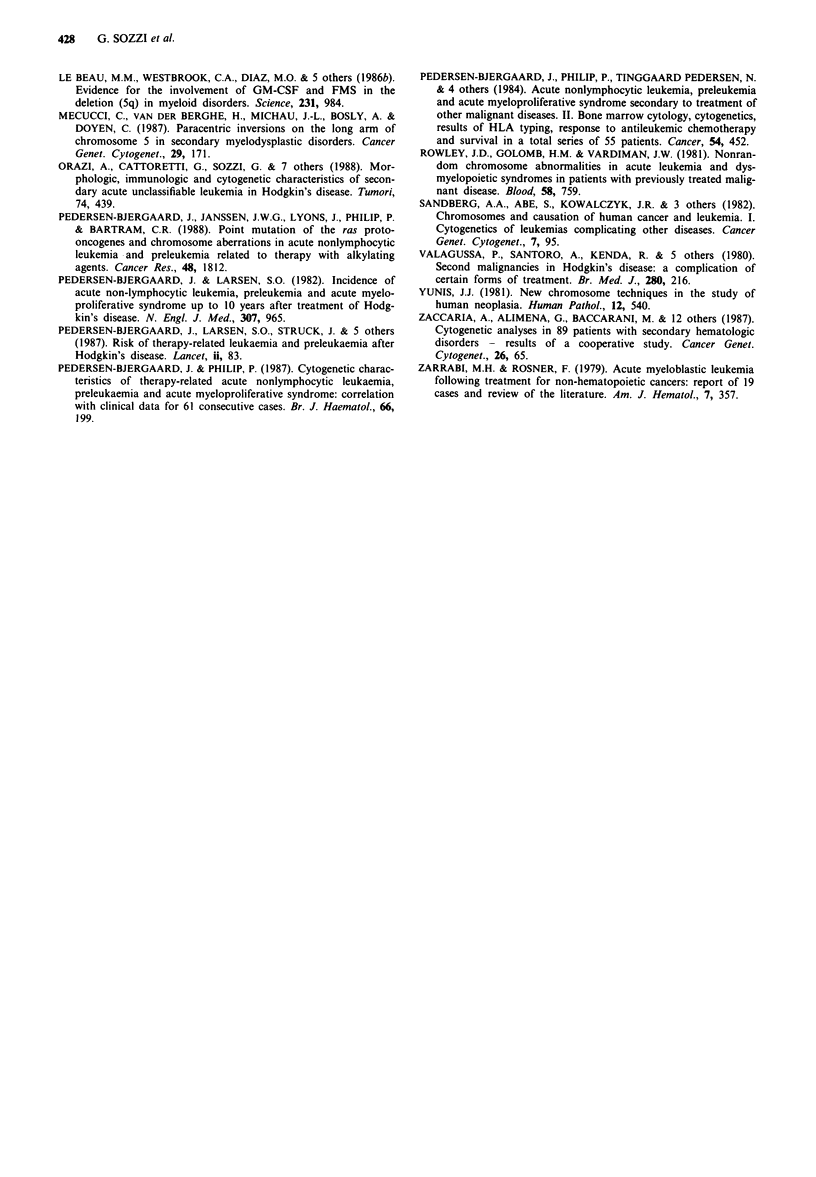# Cytogenetic study in therapy-related myelodysplastic syndromes (t-MDS) and acute non-lymphocytic leukaemia (t-ANLL).

**DOI:** 10.1038/bjc.1990.93

**Published:** 1990-03

**Authors:** G. Sozzi, M. Miozzo, A. Orazi, C. Calderone, M. Castellano, S. Viviani, A. Santoro, M. A. Pierotti, G. Della Porta

**Affiliations:** Istituto Nazionale Tumori, Milan, Italy.

## Abstract

**Images:**


© Macmillan Press Ltd., 1990

### Cytogenetic study in therapy-related myelodysplastic syndromes (t-MDS) and acute non-lymphocytic leukaemia (t-ANLL)

G. Sozzi, M. Miozzo, A. Orazi, C. Calderone, M. Castellano, S. Viviani, A. Santoro, M.A. Pierotti & G. Della Porta

*Istituto Nazionale Tumori, Via G. Venezian 1, 20133 Milan, Italy.*

**Summary** A cytogenetic study was performed in 27 patients suspected of t-MDS or t-ANLL. In 12 patients the diagnosis of t-MDS or t-ANLL was confirmed by morphological, cytochemical and immunophenotypical analysis. The cases were classified as RA (one), RAEB (four), CMML (two), ANLL (five). They had received chemotherapy and/or RT for Hodgkin's disease (eight cases), solid tumours (three cases) and multiple myeloma (one case). Clonal chromosome abnormalities were found in bone marrow or peripheral blood cells in all the 12 cases. Five patients had a clonal abnormality of chromosome no. 5 (monosomy, deletions, translocation and inversion of 5q). The critical region on chromosome no. 5 comprised bands q12-q34. Monosomy and deletion of chromosome 7q was observed in the other two patients. In the six remaining patients various karyotypic patterns were observed including a t(4;11) (q21;q23) in one case, monosomies (four cases) and trisomies (one case) of different chromosomes. In the other 15 cases, the presence of a normal karyotype together with the morphological and immunophenotypical characterisation was consistent with a diagnosis of non-neoplastic specimens.

Acute non-lymphocytic leukaemia and myelodysplastic syndrome represent severe long-term diseases following chemo/radiotherapeutic regimens for a previous tumour (Zarrabi & Rosner, 1979; Valagussa *et al.*, 1980; Anderson *et al.*, 1981; Gomez *et al.*, 1982; Pedersen-Bjergaard & Larsen, 1982; Pedersen-Bjergaard *et al.*, 1987). At the cytogenetic level the occurrence of rearranged karyotypes in bone marrow and peripheral blood cells of these patients has been consistently reported; in particular, chromosomes 5 and 7 have been shown to be significantly affected (Rowley *et al.*, 1981; Sandberg *et al.*, 1982; Pedersen-Bjergaard *et al.*, 1984, 1988; Pedersen-Bjergaard & Philip, 1987; Le Beau *et al.*, 1986*a*; Iurlo *et al.*, 1988). Complete losses or deletions of the long arm of these chromosomes have been observed and the consequent loss of gene function has been hypothesised to be crucial in the pathogenesis of these preneoplastic and overtly neoplastic forms (Le Beau *et al.*, 1986*a*, *b*).

Here we present the cytogenetic findings in 12 patients who developed therapy related myelodysplastic syndrome (t-MDS) or acute non-lymphocytic leukaemia (t-ANLL). The patients received combined treatment (chemo/radiotherapy) for Hodgkin's disease (HD) (seven cases), solid tumours (two cases) and multiple myeloma (one case). Two patients (one with HD and one with a solid tumour) were treated with RT alone. The purpose of this study was a further characterisation of the chromosome changes in these syndromes which could be useful to address molecular investigations to chromosomal regions specifically rearranged.

### Materials and methods

We investigated 27 adult patients with a previous history of chemo and/or radiotherapy for malignancy, and whose clinical and peripheral blood findings were suggestive of a diagnosis of t-MDS and/or t-ANLL. In 12 of these, the bone marrow examination confirmed the provisional diagnosis. The patients were classified according to the FAB criteria on the basis of morphological, cytochemical and cell markers analyses, as previously described (Orazi *et al.*, 1988).

In the other 15 cases the bone marrow morphology was consistent with non-myelodysplastic transient cytopenias and the peripheral blood count reverted to normality in subsequent examinations. The cytogenetic analysis was performed on bone marrow and/or peripheral blood samples using 24-48 h unstimulated cultures. Chromosome preparations were carried out according to standard methods (Yunis, 1981). At least 10 metaphases were analysed by the G-banding technique and chromosome abnormalities were described in accordance with the International System for Human Cytogenetic Nomenclature (ISCN, 1985).

### Results

Table I summarises the clinical data relative to the 12 patients. In eight cases the first tumour was diagnosed as HD whereas the other four patients have previously suffered multiple myeloma, carcinoma of the breast, basalioma and osteosarcoma, respectively. Treatment consisted of combined radiotherapy (RT) and chemotherapy except cases 5 and 12, which received only RT. The chemotherapeutic drugs always included alkylating agents.

The mean time elapsing between the beginning of treatment for primary tumour and the diagnosis of the secondary disorder was 7.8 years (range 1-12.7 years). Of the six patients who developed t-MDS, one was classified as RA according to the FAB classification, three as RAEB and two as CMML. The remaining six patients had t-ANLL of M2 FAB subtype (two cases), M5 (three cases) and AUL (one case); in two patients (nos. 11 and 12) the t-ANLL M2 was preceded by an MDS, diagnosed on bone marrow examination, with a duration of 22 and 2 months, respectively.

In two other cases (nos 8 and 9) peripheral blood anomalies preceding the onset of leukaemia and consistent with a MDS phase were observed. However, no bone marrow examinations were performed at this stage. In case nos 7 and 10, no peripheral blood disturbances were present. Mean survival time from the diagnosis of the secondary disorder was 6.4 months (range 1-18 months), with four patients still alive.

All the 15 patients who did not receive diagnosis of t-MDS or t-ANLL showed normal karyotype. The results of the cytogenetic analyses in the 12 patients with t-MDS or t-ANLL are shown in Table II. All of them presented aneuploid kayrotype with tendency to hypodiploidy. Abnormalities of chromosome 5 were observed in five patients with the complete loss of one chromosome 5 in case no. 2, a del(5) (q12q34) in cases nos 3 and 4, a t(5;20) (q12;q13) in case no. 11 and an inv5(p15.1q12) in case no. 12. Monosomy of chromosome 7 and del(7) (q22) was observed in cases nos 4 and 8, respectively. In patient no. 4, 50% of the cells analysed showed both a del(5) (q12q34) and monosomy 7.

Correspondence: G. Sozzi.
Received 1 September 1989; and in revised form 8 November 1989.

**Table I** Clinical data

| Case | Age/sex | | 1st tumour | Therapy | Interval (months) | t-MDS t-ANLL (FAB subtype) | Survival (months) |
|---|---|---|---|---|---|---|---|
| 1 | 36 | F | HD | RT, MOPP, ABVD | 126 | RA | 19 (a) |
| 2 | 59 | M | HD | RT, MOPP | 102 | RAEB | 5 (d) |
| 3 | 63 | M | multiple myeloma | RT, PRED/ADM VCR/CTX, CCNU | 56 | RAEB | 1 (d) |
| 4 | 67 | F | ca breast | RT, ADM, MMC | 93 | RAEB | 10 (d) |
| 5 | 70 | M | basalioma | RT | 60 | CMML | 28 (a) |
| 6 | 60 | M | HD | RT, MOPP, ABVD | 65 | CMML | 12 (d) |
| 7 | 32 | M | HD | RT, MOPP | 153 | AUL | 3 (d) |
| 8 | 56 | F | HD | RT, MOPP, ABVD, CEP, leukeran | 42 | RAEBt/ANLL M5 | 3 (d) |
| 9 | 32 | F | HD | RT, MOPP | 146 | RAEBt/ANLL M5 | 2 (d) |
| 10 | 17 | M | osteosarcoma | ADR, BLEO, CTX, actinomycin D | 31 | RAEBt/ANLL M5 | 24 (a) |
| 11 | 66 | F | HD | RT, MOPP | 240 | RAEBt/ANLL M2 | 25 (a) |
| 12 | 43 | M | HD | RT | 9 | RAEBt/ANLL M2 | 19 (d) |

M, male; F, female; d, died; a, alive; HD, Hodgkin's disease; RT, radiotherapy; MOPP, mechloretamina, vincristine, procarbazine, prednisone; ABVD, doxorubicin, bleomycin, vinblastine, decarbazine; PRED, prednisone; ADM, adriamycin; VCR, vincristine; CTX, cyclophosphamide; BLEO, bleomycin; CEP, CCNU, etoposide, prednimustine; CCNU, lomustine.

**Table II** Cytogenetic data

| Case | Specimen | No. of metaphases | Percentage abnormal | Karyotype |
|---|---|---|---|---|
| 1 | BM | 21 | 19 | 46xx, −12, +mar |
| 2 | BM | 10 | 50 | 46xx, −5, +mar |
| 3 | BM | 9 | 100 | 46xy,del(5)(q12q34) |
| 4 | BM | 11 | 100 | 47xx,del(5)(q12q34), +mar(50%) 46xx,same,−7(50%) |
| 5 | BM | 11 | 90 | 45xy,−22 |
| 6 | PBL | 19 | 68 | 45xy,−21 |
| 7 | PBL | 15 | 66 | 46xy,t(4;11(q21;q23) |
| 8 | BM | 10 | 100 | 46xx,del(7)(q22) (80%) 45x,same,−x (20%) |
| 9 | BM | 10 | 45 | 46x,−x, +mar |
| 10 | BM | 15 | 100 | 48xy,dup(1q)(q11→q12), +3, +9 |
| 11 | BM | 31 | 87 | 46xx,inv dup(3q)(q21→q26), t(5;20)(q12;q13) |
| 12 | BM | 15 | 100 | 46xy,inv(5)(p15.1q12),t(9;14) (p23;q21),inv(16)(p13q22) |

Partial karyotypes from patients with abnormalities of chromosome 5 and 7 are shown in Figure 1.

Monosomies of chromosomes 12, 21, 22 and X were observed in the other four cases, and in two of them the monosomy was associated with the presence of a small marker chromosome of unidentified origin. Case no. 10 presented trisomies of chromosome 3 and 6 and a marked heteromorphism between the two chromosomes 1. In case no. 7 a t(4;11) (q21;q23) was present as the only change. The immunological phenotype of this case was Tdt−, CD7−, HLA−DR+, CD19+, CD33−, suggesting an early progenitor cell bearing some lymphoid-associated antigens together with evidence of early monocytic differentiation (Orazi *et al.*, 1988).

### Discussion

In this study chromosome aberrations were observed in 100% of the patients with t-MDS and t-ANLL who had received single (RT) or combined (RT + alkylating agents) treatment for a previous neoplastic disease.

In our cases chromosome 5 was the most frequently rearranged and the abnormalities consisted in monosomy (case no. 2), del5(q12q34) (cases nos 3 and 4), and t(5;20) (q12;q13) with a derivative 5q− chromosome (case no. 11). Whereas monosomy and partial deletion of chromosome 5 has been frequently observed (Le Beau *et al.*, 1986*a*; Pedersen-Bjergaard & Philip, 1987; Pedersen-Bjergaard *et al.*, 1988; Zaccaria *et al.*, 1987; Iurlo *et al.*, 1988), the rearrangement observed in case no. 11 is, to our knowledge, the first cytogenetic evidence of a translocation of the deleted sequence from chromosome 5q to another chromosome. In addition, an inv(5) (p15.1q12) was present in case no. 12 leading to an intrachromosome relocation of the region q12-q34 of chromosome 5. Thus, this region of chromosome 5 could represent a target for mutagenic agents that might cause either complete or partial deletion of genes on 5q, or inter/intra-chromosome relocation of the region q12–q34. These observations lend support to the hypothesis that following the loss or deletion of chromosome 5q, critical gene(s) could be inactivated, resulting in alterations of cell growth control (Le Beau *et al.*, 1986*a*, *b*). In addition a number of genes coding for proteins involved in haematopoiesis are also localised on chromosome 5 (q21–q33) (GM-CSF, CSF-1, FMS, IL-3, PDGFR, ECGF, IL-5), and therefore could be directly deregulated by the observed chromosome rearrangements. The latter possibility is consistent with the karyotypes observed in our cases nos 11 and 12, which presented a translocation of the region q12-q34 and a pericentric inversion p15–q12 of chromosome 5, respectively, suggesting that also other chromosomal rearrangements could lead to an altered regulatory control due to the relocation of genes belonging to chromosome 5(q12-q34). In particular, the consistency of the breakpoints on 5q12 points to this region as being of critical importance. The same hypothesis has been suggested by Mecucci *et al.* (1987), who reported two cases of paracentric inversion of the long arm of chromosome 5 in secondary myelodysplastic syndromes.

The data relative to the prognostic value of cytogenetic findings in secondary haematological disorders are still controversial. In fact, Pedersen-Bjergaard and Philip (1987) and

**Figure 1** Partial karyotypes from patients with abnormalities of chromosomes nos 5 and 7.

Iurlo *et al.* (1988) found a significantly shorter survival in patients showing multiple chromosome aberrations as compared to patients with a single karyotypic alteration, including monosomy 7. In contrast, Le Beau *et al.* (1986*a*), in a large cytogenetic study of 63 patients with t-MDS and t-ANLL, did not find any significant difference in the clinical course and survival time when patients were grouped according to the complexity of their karyotype (abnormalities of chromosomes 5 and/or 7 alone, or with additional rearrangements). In our cases the majority of the rearranged karyotypes displayed simple chromosome changes, patient 12 being the only one with a complex karyotype. The survival time was poor in patients with abnormalities of chromosome 5 and/or 7 and in patients presenting other changes (mean values 7.2 and 5 months, respectively). However, only one of the six patients with abnormalities of chromosome 5 and/or 7 was alive, whereas three of the six patients with changes not involving these chromosomes were still alive. Thus, although the total number of patients was too small for a meaningful statistical comparison, patients with abnormalities of chromosomes 5 and/or 7 had a tendency towards a worse prognosis. It is noteworthy that the only patient alive in this group of patients (no. 11) showed a balanced translocation of 5q instead of the complete or partial deletion of this chromosome, suggesting that only the deletion of 5q and the consequent loss of critical gene(s) on 5q may be related to a worse prognosis. In addition, the other patient showing a balanced rearrangement of chromosome 5, an inv(5) (p15.1q12), without any visible loss of material on 5q (no. 12), died after the longest survival time (18 months) observed in the group of patients with abnormalities of chromosome 5 and/or 7.

In conclusion, the present results confirm that abnormalities of chromosome 5 and/or 7 are frequently observed in secondary haematological disorders and could be of diagnostic and prognostic value. Further molecular analysis of the involved chromosomal regions will clarify their role in the pathogenesis of secondary haematological disorders.

### References

ANDERSON, R.L., BAGBY, G.C., RICHERT-BOE, K., MAGENIS, R.E. & KOLER, R.D. (1981). Therapy-related preleukemic syndrome. *Cancer*, **47**, 1867.

GOMEZ, G.A., AGGARWAL, K.K. & HAN, T. (1982). Post-therapeutic acute malignant myeloproliferative syndrome and acute nonlymphocytic leukemia in non-Hodgkin's lymphoma. Correlation with intensity of treatment. *Cancer*, **50**, 2285.

ISCN (1985). An international system for human cytogenetic nomenclature. *Cytogenet. Cell Genet.*, **21**, 309.

IURLO, A., MECUCCI, C., VAN ORSHOVEN, A. & 3 others (1988). The karyotype in secondary hematologic disorders after treatment for Hodgkin's disease. A study of 19 patients. *Cancer Genet. Cytogenet.*, **36**, 165.

LE BEAU, M.M., ALBAIN, K.S., LARSON, R.A. & 5 others (1986*a*). Clinical and cytogenetic correlations in 63 patients with therapy-related myelodysplastic syndromes and acute nonlymphocytic leukemia: further evidence for characteristic abnormalities of chromosomes nos 5 and 7. *J. Clin. Oncol.*, **4**, 325.

LE BEAU, M.M., WESTBROOK, C.A., DIAZ, M.O. & 5 others (1986*b*). Evidence for the involvement of GM-CSF and FMS in the deletion (5q) in myeloid disorders. *Science*, **231**, 984.

MECUCCI, C., VAN DER BERGHE, H., MICHAU, J.-L., BOSLY, A. & DOYEN, C. (1987). Paracentric inversions on the long arm of chromosome 5 in secondary myelodysplastic disorders. *Cancer Genet. Cytogenet.*, **29**, 171.

ORAZI, A., CATTORETTI, G., SOZZI, G. & 7 others (1988). Morphologic, immunologic and cytogenetic characteristics of secondary acute unclassifiable leukemia in Hodgkin's disease. *Tumori*, 74, 439.

PEDERSEN-BJERGAARD, J., JANSSEN, J.W.G., LYONS, J., PHILIP, P. & BARTRAM, C.R. (1988). Point mutation of the *ras* protooncogenes and chromosome aberrations in acute nonlymphocytic leukemia and preleukemia related to therapy with alkylating agents. *Cancer Res.*, **48**, 1812.

PEDERSEN-BJERGAARD, J. & LARSEN, S.O. (1982). Incidence of acute non-lymphocytic leukemia, preleukemia and acute myeloproliferative syndrome up to 10 years after treatment of Hodgkin's disease. *N. Engl. J. Med.*, **307**, 965.

PEDERSEN-BJERGAARD, J., LARSEN, S.O., STRUCK, J. & 5 others (1987). Risk of therapy-related leukaemia and preleukaemia after Hodgkin's disease. *Lancet*, **ii**, 83.

PEDERSEN-BJERGAARD, J. & PHILIP, P. (1987). Cytogenetic characteristics of therapy-related acute nonlymphocytic leukaemia, preleukaemia and acute myeloproliferative syndrome: correlation with clinical data for 61 consecutive cases. *Br. J. Haematol.*, **66**, 199.

PEDERSEN-BJERGAARD, J., PHILIP, P., TINGGAARD PEDERSEN, N. & 4 others (1984). Acute nonlymphocytic leukemia, preleukemia and acute myeloproliferative syndrome secondary to treatment of other malignant diseases. II. Bone marrow cytology, cytogenetics, results of HLA typing, response to antileukemic chemotherapy and survival in a total series of 55 patients. *Cancer*, **54**, 452.

ROWLEY, J.D., GOLOMB, H.M. & VARDIMAN, J.W. (1981). Nonrandom chromosome abnormalities in acute leukemia and dysmyelopoietic syndromes in patients with previously treated malignant disease. *Blood*, **58**, 759.

SANDBERG, A.A., ABE, S., KOWALCZYK, J.R. & 3 others (1982). Chromosomes and causation of human cancer and leukemia. I. Cytogenetics of leukemias complicating other diseases. *Cancer Genet. Cytogenet.*, **7**, 95.

VALAGUSSA, P., SANTORO, A., KENDA, R. & 5 others (1980). Second malignancies in Hodgkin's disease: a complication of certain forms of treatment. *Br. Med. J.*, **280**, 216.

YUNIS, J.J. (1981). New chromosome techniques in the study of human neoplasia. *Human Pathol.*, **12**, 540.

ZACCARIA, A., ALIMENA, G., BACCARANI, M. & 12 others (1987). Cytogenetic analyses in 89 patients with secondary hematologic disorders – results of a cooperative study. *Cancer Genet. Cytogenet.*, **26**, 65.

ZARRABI, M.H. & ROSNER, F. (1979). Acute myeloblastic leukemia following treatment for non-hematopoietic cancers: report of 19 cases and review of the literature. *Am. J. Hematol.*, **7**, 357.